# A Therapeutic Strategy to Combat HIV-1 Latently Infected Cells With a Combination of Latency-Reversing Agents Containing DAG-Lactone PKC Activators

**DOI:** 10.3389/fmicb.2021.636276

**Published:** 2021-03-17

**Authors:** Kouki Matsuda, Takuya Kobayakawa, Ryusho Kariya, Kiyoto Tsuchiya, Shoraku Ryu, Kohei Tsuji, Takahiro Ishii, Hiroyuki Gatanaga, Kazuhisa Yoshimura, Seiji Okada, Akinobu Hamada, Hiroaki Mitsuya, Hirokazu Tamamura, Kenji Maeda

**Affiliations:** ^1^National Center for Global Health and Medicine Research Institute, Tokyo, Japan; ^2^Institute of Biomaterials and Bioengineering, Tokyo Medical and Dental University, Bunkyō, Japan; ^3^Division of Hematopoiesis, Joint Research Center for Human Retrovirus Infection, Kumamoto University, Kumamoto, Japan; ^4^AIDS Clinical Center, National Center for Global Health and Medicine, Shinjuku, Japan; ^5^Division of Molecular Pharmacology, National Cancer Center Research Institute, Tokyo, Japan; ^6^AIDS Research Centre, National Institute of Infectious Diseases, Tokyo, Japan; ^7^Tokyo Metropolitan Institute of Public Health, Tokyo, Japan; ^8^HIV and AIDS Malignancy Branch, National Cancer Institute, National Institutes of Health, Bethesda, MD, United States

**Keywords:** HIV-1 reservoirs, HIV-1 latently infected cells, diacylglycerol-lactone, protein kinase C activator, HIV-1

## Abstract

Advances in antiviral therapy have dramatically improved the therapeutic effects on HIV type 1 (HIV-1) infection. However, even with potent combined antiretroviral therapy, HIV-1 latently infected cells cannot be fully eradicated. Latency-reversing agents (LRAs) are considered a potential tool for eliminating such cells; however, recent *in vitro* and *in vivo* studies have raised serious concerns regarding the efficacy and safety of the “shock and kill” strategy using LRAs. In the present study, we examined the activity and safety of a panel of protein kinase C (PKC) activators with a diacylglycerol (DAG)-lactone structure that mimics DAG, an endogenous ligand for PKC isozymes. YSE028, a DAG-lactone derivative, reversed HIV-1 latency *in vitro* when tested using HIV-1 latently infected cells (e.g., ACH2 and J-Lat cells) and primary cells from HIV-1-infected individuals. The activity of YSE028 in reversing HIV-1 latency was synergistically enhanced when combined with JQ1, a bromodomain and extra-terminal inhibitor LRA. DAG-lactone PKC activators also induced caspase-mediated apoptosis, specifically in HIV-1 latently infected cells. In addition, these DAG-lactone PKC activators showed minimal toxicity *in vitro* and *in vivo*. These data suggest that DAG-lactone PKC activators may serve as potential candidates for combination therapy against HIV-1 latently infected cells, especially when combined with other LRAs with a different mechanism, to minimize side effects and achieve maximum efficacy in various reservoir cells of the whole body.

## Introduction

Although prolonged combination antiretroviral therapy (cART) has succeeded in reducing HIV type 1 (HIV-1) replication, the virus cannot be completely eradicated from the bodies of people living with HIV-1 (PLWH) because of the persistent latently infected cells located in viral reservoirs, or so-called sanctuaries, in the body (Siliciano et al., 2003; Cillo et al., 2014). At present, a novel approach to eradicate these reservoir cells using latency-reversing agents (LRAs), which are small-molecule agents, called “shock and kill” is being considered (Hamer, 2004; Richman et al., 2009). However, recent clinical trials demonstrated no reduction in HIV-1 reservoir cells using LRAs *in vivo*, despite the fact that these drugs are active *in vitro* (Archin et al., 2012; Rasmussen et al., 2014). Furthermore, it is necessary to develop agents with reduced toxicity because most LRA candidates act through host cells rather than viruses.

Recent studies have reported that many small-molecule compounds, including histone deacetylase (HDAC) inhibitors, bromodomain and extra-terminal (BET) inhibitors, and protein kinase C (PKC) agonists, show HIV-1 latency-reversing activity (Contreras et al., 2009; Boehm et al., 2013; Jiang et al., 2015). PKC isozymes are a family of serine-threonine kinases, consisting of several isozymes that play a role in physiological cellular responses (Nishizuka, 1992; Watanabe et al., 1992; Mischak et al., 1993; Li et al., 2003; Martin-Diaz et al., 2007). PKC isozymes are divided into three subfamilies: conventional PKCs (cPKCs: α, β, and γ), novel PKCs (nPKCs: δ, ε, η, and θ), and atypical PKCs (aPKCs: ι and ζ; Griner and Kazanietz, 2007). cPKCs and nPKCs are regulated by ligand binding through their tandem C1 domains (C1a and C1b), with the exception of aPKCs. Additionally, cPKCs require binding of Ca^2+^ to the C2 domain. PKCs are located in the cytosol in the inactive form, and substrate binding is capped by its own pseudosubstrate. This form translocates from the cytosol to the plasma membrane and internal membranes, followed by ligand binding. The endogenous ligand of PKCs is a second messenger, 1,2-diacylglycerol (DAG), which is generated downstream of receptor tyrosine kinases and G-protein coupled receptors (Kishimoto et al., 1980). DAG is produced at the inner face of the plasma membrane, and its binding to the C1 domain induces a conformational change of PKCs into the active form (Ogawa et al., 1981; Sakai et al., 1997). Binding subsequently causes the translocation of PKCs into the plasma membrane, followed by signaling through multiple downstream pathways (Wang, 2006). DAG-lactone derivatives, which function as conformationally constrained DAG analogs, are synthetically tractable and have been structurally optimized to improve PKC binding affinity and selectivity between classes of C1 domain-containing targets (Tamamura et al., 2000; Ohashi et al., 2017).

In this study, we focused on the HIV-1 latency-reversing activity and safety of DAG-lactone derivatives, including YSE028 (Nomura et al., 2011), which exhibited a potent ability to activate latent HIV-1 infected cells without any toxicity *in vitro* and *in vivo*.

## Materials And Methods

### Drugs and Reagents

**Figure 1:**
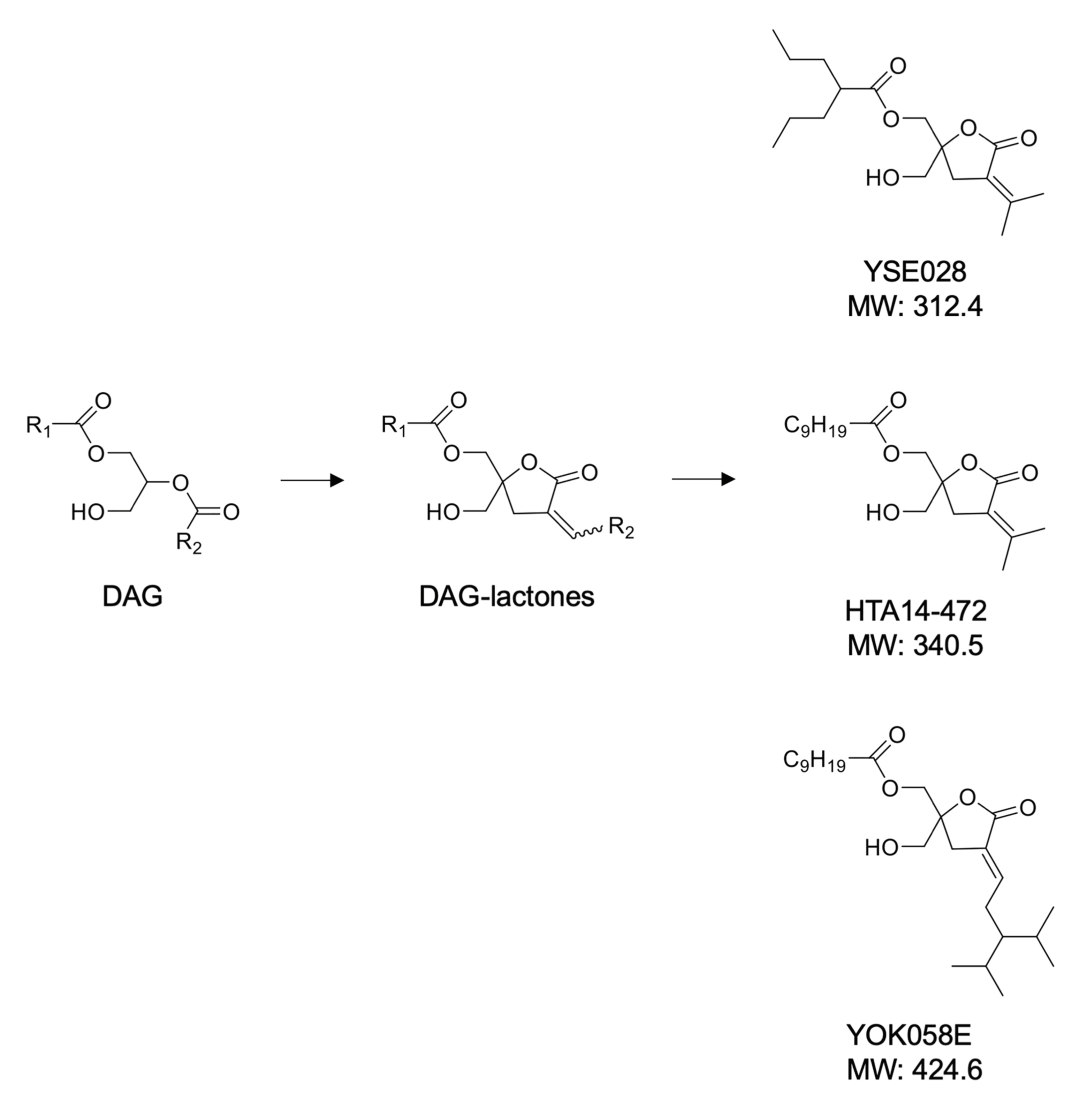
Structures of diacylglycerol (DAG)-lactone derivatives.

A panel of DAG-lactone derivatives, including YSE028, was synthesized as described previously (Nacro et al., 2000; Tamamura et al., 2000; Nomura et al., 2011; Figure 1A). Prostratin (PKC activator), PEP005 (PKC activator), and JQ-1 (BET inhibitor) were purchased from Sigma-Aldrich (St. Louis, MO, United States), Cyman Chemical (Ann Arbor, MI, United States), and BioVision (Milpitas, CA, United States), respectively. Phorbol 12-myristate 13-acetate (PMA) was purchased from Wako Pure Chemical (Osaka, Japan).

### Cells

Two latent HIV-1 infected cell lines, ACH-2 cells derived from the A3.01 cell line, and U1 cells derived from the U937 promonocytic cell line, were used in this study. J-Lat10.6 cells derived from Jurkat T cells were also used. These cell lines were obtained from the National Institutes of Health (NIH) AIDS Reagent Program. Cells were maintained in RPMI1640 medium (Sigma-Aldrich) supplemented with 10% fetal bovine serum (FBS, Sigma-Aldrich), 50 U/ml penicillin, and 50 μg/ml kanamycin. In experiments (Supplementary Figure S4), heat-inactivated (56°C, 30 min) mouse serum from BALB/c mice was used instead of FBS.

### HIV-1 Latency Reversal With LRAs

The reactivation of HIV-1 from latently infected cells was determined by intracellular p24 protein expression and quantification of p24 antigen in the supernatant (ACH-2 and U1 cells), or by changes in intracellular green fluorescent protein (GFP) expression (J-Lat 10.6 cells). J-Lat (Jordan et al., 2003), ACH-2, or U1 cells (5 × 10^5^ cells/ml) were seeded in 96-well plates and incubated with different drug concentrations for 24 h to collect the cells. The supernatant was collected after 48 h. The increase in supernatant p24 antigen levels was measured using a Lumipulse G1200 (FUJIREBIO, Tokyo, Japan). J-Lat cells (5 × 10^5^ cells/ml) were placed in 48-well plates and incubated with different drug concentrations for 24 h. Intracellular p24 protein expression and GFP-positive cells were analyzed by flow cytometry.

### Cytotoxicity Assays

To determine the cytotoxicity of LRAs, A3.01, and U937 cells (5 × 10^5^ cells/ml), which are the parental cells for ACH-2 and U1 cells, respectively, were cultured in the presence or absence of LRAs. After 7 days, cell viability was examined using the Cell Counting Kit-8 assay (Dojindo, Kumamoto, Japan) according to the manufacturer’s instructions. The numbers of living cells after drug treatment were measured and compared to those in untreated cells and are presented as a percentage relative to the control.

### Primary CD4^+^ T Cell Isolation From HIV-1^+^ Individuals and *ex vivo* Reactivation

Primary cells were isolated from seven HIV-1^+^ individuals, and *ex vivo* reactivation experiments were conducted as previously described (Matsuda et al., 2019). In brief, peripheral blood samples were collected from HIV-1-infected participants receiving cART for at least 5 years (Table 1). All subjects maintained a low viral load (<20 copies/ml, except for occasional “blips”) during therapy. CD4^+^ T cell counts in peripheral blood samples ranged from 477 to 992 cells/mm^3^ (average: 631 cells/mm^3^), and plasma viral loads were <20 copies/ml (except for one participant whose viral load was 22 copies/ml) as measured by quantitative PCR (qPCR; COBAS AmpliPrep/COBAS TaqMan HIV-1 Test version 2.0; Roche Diagnostics, Basel, Switzerland) at the time of study enrollment. The Ethics Committee at the National Center for Global Health and Medicine approved this study (NCGM-G-002259-00), and each patient provided written informed consent. Whole peripheral blood mononuclear cells were separated by density gradient centrifugation using Ficoll-Paque™ (GE Healthcare, Munich, Germany), and CD4^+^ T cells were purified using the MojoSort™ Human CD4 T Cell Isolation Kit (BioLegend, San Diego, CA, United States) according to the manufacturer’s instructions. Purified CD4^+^ T cells were plated at a density of >2.0 × 10^6^ cells/ml and treated with 100 nM PMA, 2 μM ionomycin, 10 μM YSE028, 1 μM JQ1, or a combination for 24 h, and the cells were collected for RNA purification. For the no-drug control, the same volume of PBS (solvent used for the drugs) was added to the wells. Total RNA was extracted using an RNeasy Mini Kit (Qiagen, Hilden, Germany), following the manufacturer’s protocol. Real-time qPCR (RT-qPCR) for intracellular HIV-1 RNA was then performed using the One Step PrimeScript III RT-qPCR Mix (Takara Bio, Shiga, Japan) according to the manufacturer’s instructions. The oligonucleotide primers used were as follows: 5'-TGTGTGCCCGTCTGTTGTGT-3' (forward), 5'-GAGTCCTGCGTCGAGAGAGC-3' (reverse), and 5'-FAM-CAGTGGCGCCCGAACAGGGA-BHQ1-3' (probe) for HIV-1 RNA detection. HIV-1 RNA copy numbers were normalized to RNA input (Jiang et al., 2015). In this method, as the reduction of cell numbers due to the toxicity of a drug results in a relatively higher HIV-RNA count, we examined and confirmed that the drug did not induce toxicity in primary cells at the tested concentrations (data not shown). The number of HIV-1 RNA copies was calculated using a standard curve obtained from serially diluted HIV-1_pNL4-3_ plasmid, and normalized values [HIV-1 RNA copies/input RNA (ng)] for each drug were compared to those without drug treatment. The relative increase in HIV-1 RNA levels in the presence of each drug or combination was then determined.

**Table 1:** Clinical characteristics of HIV^+^ participants of this study.

Participant	M/F	Age	VL^a^ (copies/ml)	CD4 count^a^ (cells/mm^3^)	cART^b^	Therapy (years)	Plasma HIV RNA <20 copies/ml for (years)
Participant 1	M	47	<20	992	FTC/TAF/COBI/EVG	22	8
Participant 2	M	54	<20	753	FTC/TAF/COBI/DRV	14	8
Participant 3	M	48	22	531	FTC/TAF/DTG	17	8
Participant 4	M	55	<20	477	FTC/TAF/DTG	22	8
Participant 5	M	59	<20	587	FTC/TAF/RPV	23	8
Participant 6	M	53	<20	536	FTC/TAF/COBI/EVG	23	8
Participant 7	M	51	<20	540	FTC/TAF/COBI/EVG	15	7

a VL and CD4 count: at the time of the study.

b COBI, cobicistat; DRV, darunavir; EVG, elvitegravir; DTG, dolutegravir; FTC, embricitabine; RPV, rilpivirine; TAF, tenofovir alafenamide fumarate.

### Flow Cytometry Analysis

The amount of intracellular HIV-1 p24 and the active form of caspase-3 were determined by flow cytometry, as previously described (Matsuda et al., 2015, 2019). In brief, ACH-2 and U1 cells (2.5 × 10^5^ cells/ml) were fixed with 1% paraformaldehyde/PBS for 20 min and permeabilized with Flow Cytometry Perm Buffer (TONBO Biosciences, San Diego, CA, United States). After 5 min of incubation at room temperature, the cells were stained with FITC anti-HIV-1 p24 (24-4) monoclonal antibody (mAb; Santa Cruz Biotechnology, Dallas, TX, United States) or Alexa Fluor 647-conjugated anti-active caspase-3 (C92-605) mAb (BD Pharmingen, San Diego, CA, United States) for 30 min on ice. For T cell activation and exhaustion marker staining, PBMCs from healthy donors separated by the above density gradient centrifugation method were incubated with fixable viability stain Ghost Dye 780 (TONBO Biosciences) for 30 min on ice. The cells were then stained with Brilliant Violet 510 anti-human CD3 (UCHT1) mAb (BioLegend), FITC anti-human CD4 (RPA-T4) mAb (TONBO Biosciences), PE-Cy7 anti-human CD8a (RPA-T8) mAb (TONBO Biosciences), PerCP-Cy5.5 anti-human CD38 (HB-7) mAb (BioLegend), PE anti-human CD69 (FN50) mAb (BioLegend), or Alexa Fluor 647 anti-human CD279/PD-1 (EH12.1) mAb (BD Biosciences, San Jose, CA, United States) for 30 min on ice. Next, the cells were analyzed using BD FACSVerse (BD Biosciences). In the analysis, unstained cells were used as a negative control to set the gating for each experiment. The collected data were analyzed using FlowJo software (Tree Star, San Carlos, CA, United States).

### *In vivo* Toxicity

BALB/c mice were purchased from Charles River Laboratories Japan, Inc. (Yokohama, Japan). Female mice at 5 weeks of age were used for the experiments. The mice were housed and monitored in our animal research facility according to the institutional guidelines. All experimental procedures and protocols were approved by the Institutional Animal Care and Use Committee of Kumamoto University. PEP005 and YSE028 were dissolved in 1% dimethyl sulfoxide (DMSO) containing RPMI 1640. PEP005 (100, 300, and 1,000 μg/kg), YSE028 (100, 300, 1,000, 3,000, and 10,000 μg/kg), or 1% DMSO containing RPMI 1640 were intraperitoneally injected into BALB/c mice. After 24 h, the survival rate was determined.

### Pharmacokinetics in Mice and LC-MS/MS Analysis

Blood was drawn from mice at 0.5, 1, 3, 6, 12, and 24 h after subcutaneous administration of YSE028 at a dose of 10 mg/kg. Plasma samples were prepared on ice. Blood was collected in a heparin tube and then separated at 5,000 rpm for 10 min at 4°C. Two hundred microliter of MeOH was added to 40 μl of mouse plasma immediately after plasma collection to terminate the enzymatic hydrolysis of YSE028 during sample preparation. All samples were vortexed for 10s and centrifuged at 12,000 *g* for 10 min at 4°C. The supernatant was then separated. Alectinib (10 ng/ml) in MeOH was used as an internal standard (IS) and added to the supernatant for liquid chromatography tandem mass spectrometry (LC-MS/MS) analysis.

A Nexera X2 UHPLC system (Shimadzu, Kyoto, Japan) connected to a QTRAP5500 mass spectrometer (AB SCIEX, Framingham, MA, United States) was used for LC-MS/MS analysis. The separation of YSE028 and alectinib (IS) was performed using an XBridge C18 column (2.1 × 50 mm, 3.5 μm, Waters). The mobile phases consisted of 10 mM ammonium formate (A) and MeOH (B). The run time was 6 min with a flow rate of 0.5 ml/min. The oven temperature was set to 40°C, and the autosampler chamber was maintained at 4°C. The gradient elution program was set as follows: 0–3.0 min, 60–80% B; 3.1 min–4.0 min, 95% B; and 4.1 min–6 min, 60% B. The sample injection volume was 5 μl. The electrospray ionization interface in positive mode was used to perform tandem mass spectrometry (MS/MS). The MRM transition for YSE028 was m/z 313.1→187.1. The MS parameters were optimized as follows: desolvation temperature, 500°C; curtain gas (CUR), 40; collision gas (CAD), 7; ionspray voltage (IS), 4500v; ion source gas (GS1), 50 psi; ion source gas (GS2), 80 psi; declustering potential (DP), 110 v; entrance potential (EP), 10v; collision energy (CE), 13v; collision cell exit potential (CXP), 20v.

### Statistical Analysis

Differences between groups were analyzed for statistical significance using unpaired *t*-tests. Statistical significance was set at *p* < 0.05. Statistical analyses were performed using GraphPad Prism version 8 (GraphPad Software, La Jolla, CA, United States). CompuSyn software, based on the quantitative analysis of dose-effect relationships between multiple drugs by Chou and Talalay, was utilized to determine the synergism or antagonism of drug combinations (Chou and Talalay, 1984). To confirm synergy, this software calculates combination index (CI) values, which further assist in determining the nature of the combination compared to single drug effects. A CI value <1 indicates synergistic effects.

## Results

### Ability of DAG-Lactone Derivatives to Reverse HIV-1 Latency

**Figure 2:**
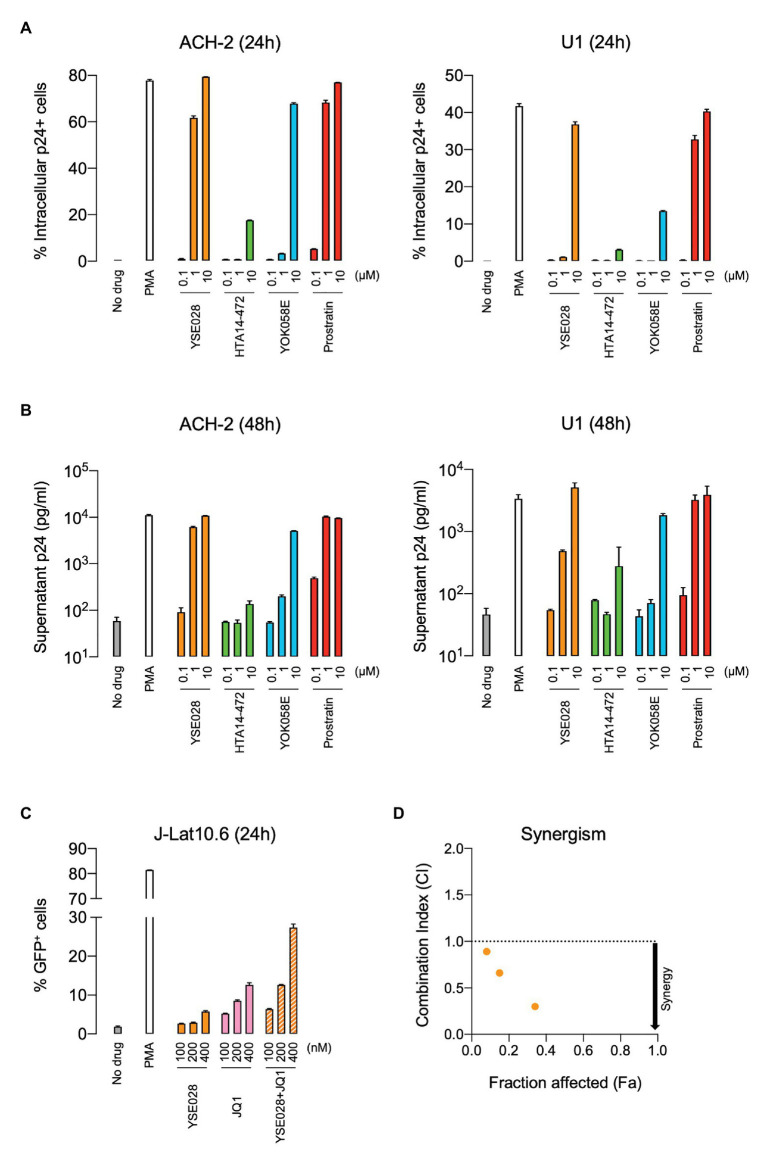
Reversal of HIV-1 latency with DAG-lactone derivatives *in vitro*. ACH-2 and U1 cells were exposed to a DAG-lactone derivative and prostratin. The expression of intracellular HIV-1 p24 protein **(A)** and production of p24 in the supernatant **(B)** were measured after 24 and 48 h of incubation, respectively. **(C)** J-Lat 10.6 cells were exposed to different concentrations of YSE028 or JQ1 or a combination of both, and the change in the number of green fluorescent protein (GFP)-positive cells was analyzed after 24 h by flow cytometry. **(D)** Synergism in drug combinations was examined using CompuSyn software. Combination index (CI) values <1 indicate synergistic effects. Data are shown as means ± standard deviations of three independent experiments.

The structures of the DAG-lactone derivatives employed in this study are shown in Figure 1. First, we determined the cytotoxicity of these compounds in A3.01 and U937 cells, which are the parental cell lines to HIV-1 latently infected ACH-2 cells and U1 cells, respectively. None of the DAG-lactone derivatives, nor another PKC agonist, prostratin, showed cytotoxicity at a maximum concentration of 100 μM (Table 2; Supplementary Figures S1A,B). We then examined HIV-1 latency reversal activity. As shown in Figure 2, all DAG-lactone derivatives induced an increase in intracellular p24^+^ cell numbers (Figure 2A; Supplementary Figure S2) or viral production (Figure 2B) in ACH-2 cells and U1 cells. The half-maximal effective concentration (EC_50_) values were calculated using the maximum viral production level determined in the presence of 10 nM PMA as 100% in ACH-2 and U1 cells (Table 3). Notably, YSE028 showed the most potent LRA activity in ACH-2 cells and U1 cells (EC_50_: 0.87 and 1.81 μM, respectively; Table 3).

**Table 2:** Cytotoxicity of latency-reversing agents (LRAs) in A3.01 and U937 cells.

CC_50_ (μM)		
Compound	A3.01	U937
YSE028	>100.0	>100.0
HTA14-472	>100.0	>100.0
YOK058E	>100.0	>100.0
Prostratin	>100.0	>100.0

Cell viability was determined using the 3-(4,5-dimethylthiazol-2-yl)-2,5-diphenyltetrazolium assay on day 7. CC_50_, the compound concentration required to reduce the viability of parental cells by 50%.

**Table 3:** Latency-reversing agent activity of the tested compounds in ACH-2 and U1 cells.

EC_50_ (μM)		
Compound	ACH-2	U1
YSE028	0.87	1.81
HTA14-472	>10.0	>10.0
YOK058E	>10.0	8.24
Prostratin	0.33	0.32

The magnitude of reactivation induced by 10 nM PMA was defined as 100% reactivation, and the concentration of each compound resulting in 50% reactivation (viral production) was used to define the EC_50_ values.

Previous *in vitro* studies have shown the importance of the combined use of LRAs with different classes to achieve higher levels of HIV-1 reversal activity (Laird et al., 2015). In fact, the combination of a PKC agonist (e.g., PEP005 and benzolactam derivatives) and a BET inhibitor, JQ1, exhibited synergism in HIV-1 reversal activity (Jiang et al., 2015; Matsuda et al., 2019). Thus, we examined the effect of the combination of YSE028 and JQ1 on HIV-1 reversal in J-Lat 10.6 cells. J-Lat 10.6 cells contain a full-length HIV-1 genome with non-functional *Env* due to a frameshift and GFP in the *Nef* region, which enables the detection of HIV-1 reversal as increasing GFP expression levels (Jordan et al., 2003). As shown in Figure 2C, treatment with YSE028 or JQ1 (100–400 nM) increased the number of GFP^+^ cells to 5.8 and 12.6%, respectively. When cells were treated with the combination, the number of GFP^+^ cells increased to 27.4% (Figure 2C). The effects of the combination were analyzed using Compusyn software, and it was found that the combination of YSE028 and JQ1 had a synergistic effect at 100, 200, and 400 nM (CI = 0.89, 0.66, and 0.30, respectively; Figure 2D).

### YSE028 Reverses HIV-1 Latency in Primary CD4^+^ T Cells From HIV-1-Infected Individuals *ex vivo*

**Figure 3:**
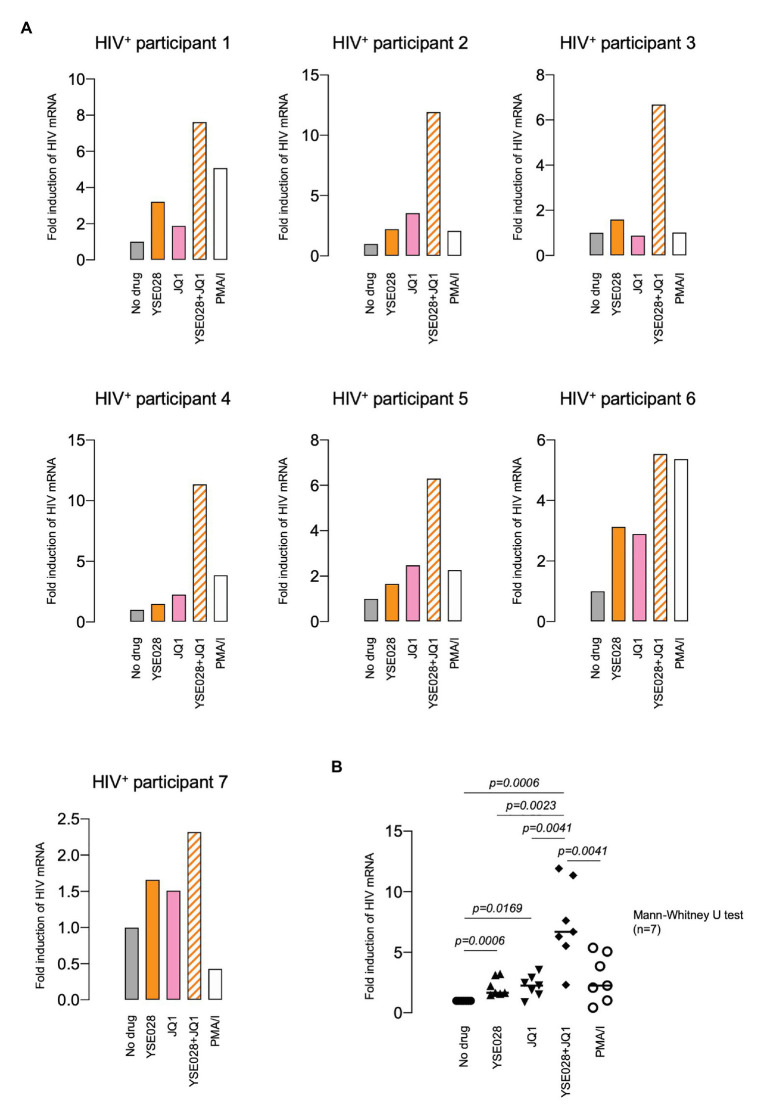
YSE028 reactivates HIV-1 in CD4^+^ T cells from HIV-1-infected individuals. **(A)** Human CD4^+^ T cells purified from seven HIV-1-infected individuals undergoing cART (Table 1) were treated with 10 μM YSE028, 1 μM JQ1, a combination of YSE028 and JQ1, or 100 nM PMA plus 2 μM ionomycin for 24 h. Intracellular HIV-1 mRNA levels were detected by quantitative real-time PCR (qRT-PCR) and compared to those in untreated controls. **(B)** Statistical significance was determined using a Mann-Whitney U test, where a value of *p* < 0.05 was considered to be significant.

We then examined the effect of YSE028 using primary isolated CD4^+^ T cells from seven HIV-1-infected individuals undergoing treatment with cART (Table 1). CD4^+^ T cells in the peripheral blood of HIV-1-infected individuals were treated with 10 μM YSE028, 1 μM JQ1, and 100 nM PMA plus 2 μM ionomycin for 24 h, harvested, and HIV-1 mRNA levels in the cells were evaluated. As shown in Figure 3A, singular treatment with YSE028 enhanced HIV-1 mRNA transcription in CD4^+^ T cells in all seven individuals (1.66-fold compared to no drug control; *p* = 0.0006), and JQ1 alone also enhanced HIV-1 mRNA in six out of seven donors (2.25-fold compared to no drug control; *p* = 0.0169). In contrast, a combination of YSE028 and JQ1 significantly enhanced the level of HIV-1 mRNA (6.69-fold compared to no drug control; *p* = 0.0006), and the effect of the combination on LRA activity was significant when compared with YSE028 or JQ1 singular treatment (*p* = 0.0023 and 0.0041, respectively; Figures 3A,B). It is possible that the combination of LRAs of different classes causes an unexpected increase in toxicity in primary cells. Thus, we investigated the toxicity of LRAs in combination and found that YSE028 did not cause increased toxicity in combination (Supplementary Figure S3A).

### DAG-Lactone Derivatives Induce Apoptotic Cell Death in HIV Latently Infected Cells *via* Caspase-3 Activation

**Figure 4:**
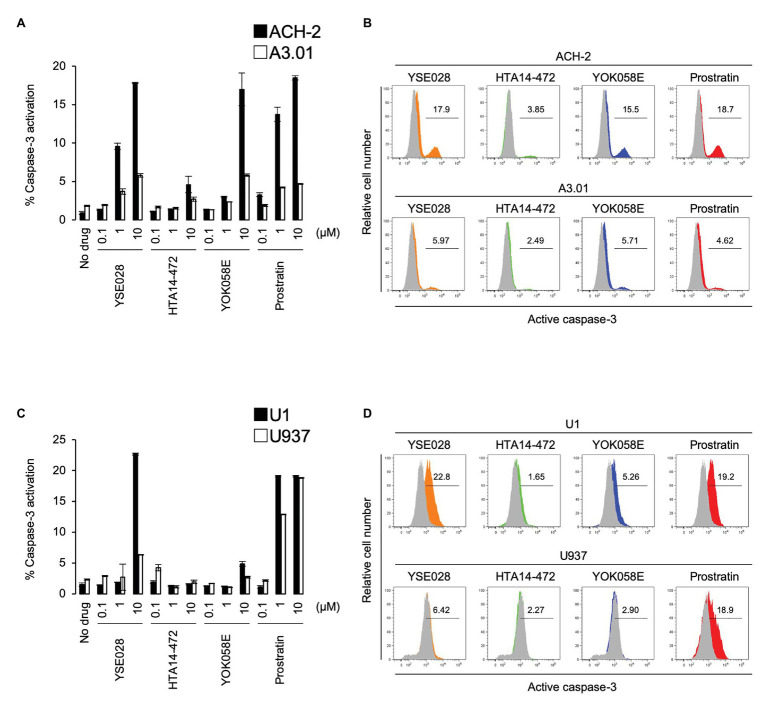
Diacylglycerol-lactone derivatives specifically induce caspase-3 activation in HIV-1 latently infected cells. The active form of caspase-3 was measured by flow cytometry. **(A)** ACH-2 and A3.01 cells were exposed to different concentrations of DAG-lactone derivatives and prostratin for 24 h. **(B)** The histogram shows representative data for caspase-3 activation with exposure to 10 μM reagent in ACH-2 and A3.01 cells. **(C)** U1 and U937 cells were exposed to different concentrations of DAG-lactone derivatives and prostratin for 24 h. **(D)** The histogram shows representative data for caspase-3 activation with exposure to 10 μM reagent in U1 and U937 cells. Data are shown as means ± SDs of three independent experiments.

In theory, reactivated HIV-1 latently infected cells treated with an LRA are eliminated by host immune systems, such as cytotoxic T lymphocytes (CTL), which is the major mechanism for reducing HIV-1 reservoir cells in the “shock and kill” strategy. However, apoptosis and cell death by the viral cytopathic effect in reactivated cells is regarded as another important mechanism to reduce HIV-1 reservoir cells *in vivo* (Hattori et al., 2018; Kim et al., 2018). Therefore, we determined the effects of DAG-lactone derivative-induced apoptosis in ACH-2 cells and U1 cells and compared them with those in their corresponding parental cells. The cells were treated with a compound (100 nM to 10 μM) for 24 h, and active caspase-3 expression levels were measured by flow cytometry. Among these derivatives, YSE028 had the greatest apoptotic effect in HIV-1 latently infected cells (17.85 and 22.7% caspase-3 activation at 10 μM in ACH-2 and U1 cells, respectively). Whereas, it showed minimal caspase-3 induction in their parental cells (5.81 and 6.35% caspase-3 activation at 10 μM in A3.01 and U937 cells, respectively; Figure 4). Interestingly, prostratin induced nearly the same levels of caspase-3 activation in U1 and its parental U937 cells (19.1 and 18.8% caspase-3 activation at 10 μM, respectively; Figures 4C,D), suggesting that the apoptosis induced in U1 cells by prostratin was not specific to HIV-1 latently infected cells. Taken together, DAG-lactone derivatives, including YSE028, appear to induce greater apoptosis in HIV-1 latently infected cells than in uninfected cells. In the experiment shown in Figure 4, we used higher concentrations (e.g., 10 μM) of PKC activators to elicit apoptosis. As shown in Supplementary Figures S1A,B, YSE028 showed only moderate toxicity in cell lines; however, it is likely that the immortalized cell lines have different toxicity profiles than human primary cells. Thus, we examined the toxicity of YSE028 at higher concentrations in primary cells and found that PKC activators, including YSE028, did not show acute toxicity up to 100 μM (Supplementary Figure S1C).

**Figure 5:**
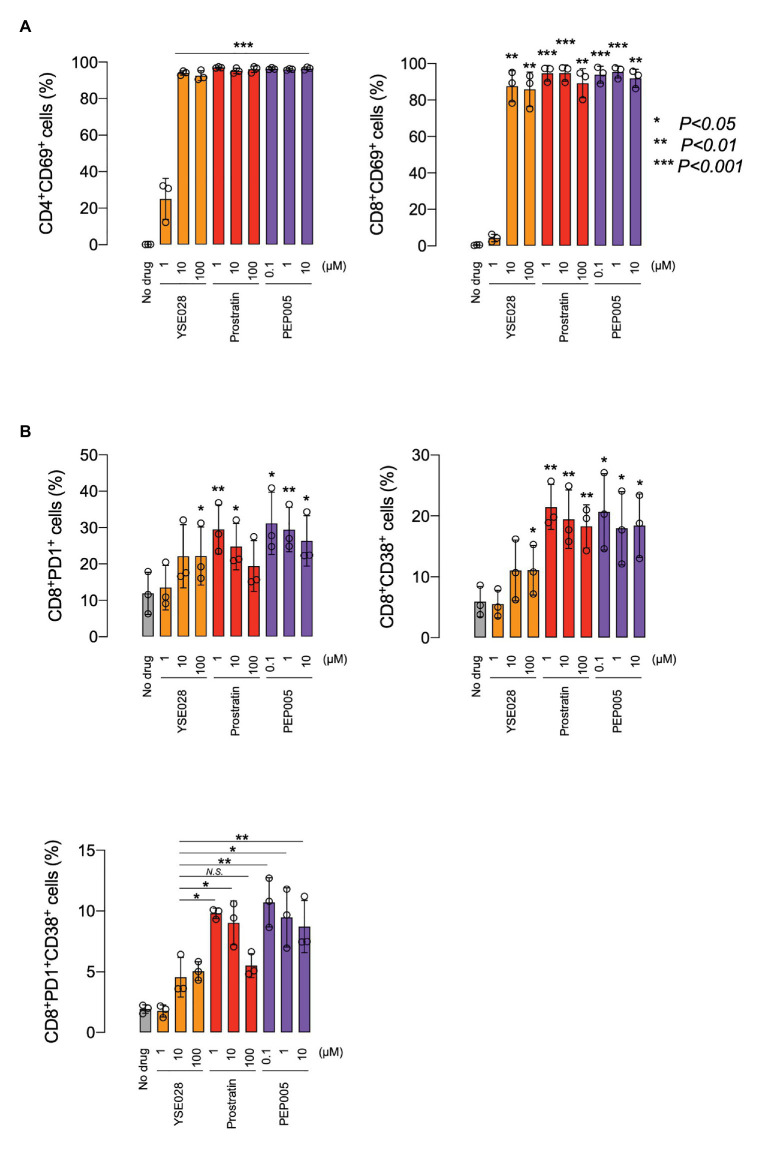
The effect of YSE028 on T cell activation. PBMCs from three healthy donors were exposed to different concentrations of a reagent for 24 h. Changes in CD69 expression on CD4^+^ or CD8^+^ primary T cells **(A)**, CD8^+^PD1^+^ primary T cells, CD8^+^CD38^+^ primary T cells, and CD8^+^PD1^+^CD38^+^ primary T cells **(B)** were analyzed by flow cytometry. Data are shown as means ± SDs. Statistical significance was determined using a paired T-test, where a value of *p* < 0.05 was considered to be significant.

Because prostratin had a different profile in inducing caspase 3 activation in U937 cells, we performed experiments to investigate the difference in the activation profile of PKC activators. Figure 5A shows the changes in the ratio of CD69^+^ cells in primary cells, which is one of the markers for global T cell activation. While YSE028 had less effect at 1 μM compared to other PKC activators (prostratin and PEP005), all tested compounds at 10 μM or more completely activated CD4^+^ and CD8^+^ T cells. The combination of YSE028 and JQ1 also elevated the expression of CD69 in primary T cells (Supplementary Figure S3B).

In HIV-1 infected individuals, the increase in CD8^+^ PD1^+^ CD38^+^ T cells is thought to be associated with immune exhaustion and disease progression (Resino et al., 2004; Trautmann et al., 2006). Recently, it has been reported that the administration of certain LRAs (e.g., HDAC inhibitor) impairs CTL-mediated IFN-γ production, which results in dysfunctional immunological responses for clearance of HIV-1 reservoir cells (Jones et al., 2014). In this study, we investigated the effect of PKC activators on CD8^+^ PD1^+^ CD38^+^ T cells, which are indicators of immune exhaustion, and found that a significant increase in CD8, PD1, and CD38 was observed in cells treated with prostratin and PEP005, while the increase with YSE028 was only moderate (Figure 5B). The mechanism underlying this difference is still unknown, but the results suggest that the profiles of T cell activation differ depending on the PKC activator.

### *In vivo* Cytotoxicity of YSE028

**Figure 6:**
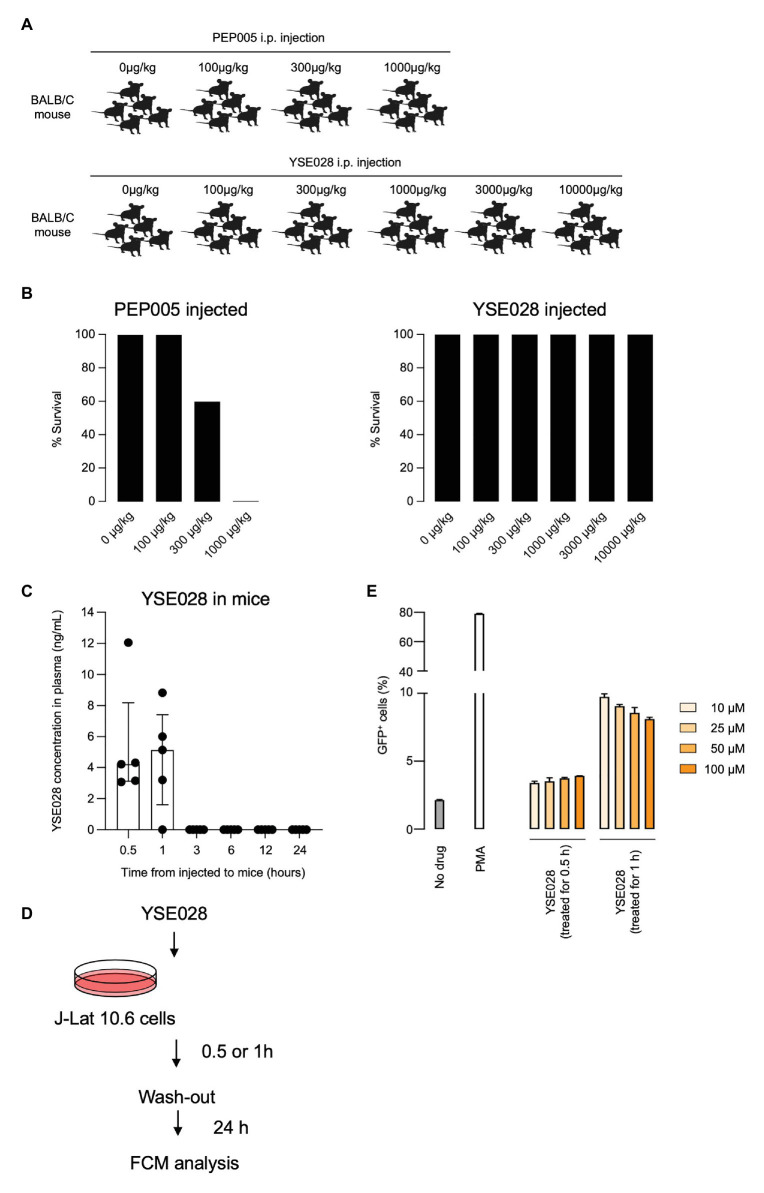
*In vivo* toxicity and pharmacokinetic analyses of YSE028. **(A)** The experimental scheme is illustrated. BALB/c mice were challenged with increasing concentrations of PEP005 or YSE028 by intraperitoneal injection, with five animals in each group. **(B)** The survival rate of PEP005- and YSE028-injected mice. **(C)** Plasma concentration of YSE028 in BALB/c mice. The concentrations were measured by LC-MS/MS at 0.5, 1, 3, 6, 12, and 24 h after administration of YSE028 at a dose of 10 mg/kg. Data are shown as medians with interquartile ranges, *n* = 5. **(D)** The experimental scheme for short-term exposure to YSE028. **(E)** J-Lat 10.6 cells were exposed to YSE028 for 0.5 or 1 h, and then reagent was washed-out. The number of GFP-positive cells was analyzed after 24 h by flow cytometry. Data are shown as means ± SDs of three independent experiments.

Finally, we tested the *in vivo* safety of the administration of a DAG-lactone derivative, YSE028, which showed potent LRA activity. PEP005, a PKC activator, was tested as a control. In the acute toxicity tests (for 24 h) with exposure to a single drug in BALB/c mice, groups of five animals were challenged with increasing concentrations of a compound by intraperitoneal injection (Figures 6A,B). PEP005 started to show acute toxicity in mice at a concentration of 300 μg/kg, and all tested mice died at 1,000 μg/kg. On the other hand, none of the animals injected with YSE028 (100 μg/kg–10 mg/kg) displayed any abnormalities in their condition as determined by their weight and fur texture (data not shown), and all of them survived (Figures 6A,B).

Because YSE028 showed no toxicity in mice at extremely high concentrations, we investigated the pharmacokinetics of YSE028 in mice. YSE028 (10 mg/kg) was administered subcutaneously to each mouse, blood was drawn at each data point, and their concentrations were measured. As shown in Figure 6C, the peak concentration was 5.14 ng/ml (median) at 1 h, and the concentration of YSE028 in the blood decreased rapidly (within 3 h; Figure 6C). We speculate that YSE028 disappeared from mouse blood because it is metabolized by esterases such as mouse carboxylesterase 1 (CES1). It is known that large amounts of CES1 are present in mouse or rat blood, but not in human blood (Hosokawa, 2008; Di, 2019). Thus, we examined the LRA activities of YSE028 and prostratin in the presence of 10% FBS or 10% mouse serum. The reactivation level of prostratin did not change in either condition, whereas the activity of YSE028 drastically decreased in the presence of mouse serum (Supplementary Figure S4). Thus, it is possible that the kinetics of DAG-lactone derivatives in humans may be different from those in mice.

To examine whether LRAs can show activity with such short exposure to HIV-1 latent cells, we conducted an *in vitro* experiment. J-Lat cells were exposed to YSE028 for 0.5 or 1 h, rinsed, and incubated for 24 h, and then the reactivation in cells was determined (Figure 6D). We found that exposure to YSE028 for 1 h successfully reactivated HIV-1-latently infected J-Lat cells (Figure 6E). Taken together, these results indicate that short-term exposure to LRAs may be a good strategy for reactivating HIV-1-latently infected cells with less toxicity *in vivo*.

## Discussion

In this study, we demonstrated the potential for HIV-1 reversal by DAG-lactone derivatives as LRA candidates. Previous studies have reported many candidate small-molecule compounds, including HDAC inhibitors, BET inhibitors, and PKC agonists (Contreras et al., 2009; Boehm et al., 2013; Jiang et al., 2015). Among them, PKC agonists have the most potent activity, but they also exert unexpected side effects. In particular, PKC activation has been reported to lead to global T-cell activation and toxicity. The classic PKC agonist phorbol ester PMA displays tumor-promoting activity that can be of crucial significance to immunodeficient patients. DAG-lactone derivatives mimic the endogenous second messenger DAG of PKC isoforms that play a role in physiological cellular responses (Nishizuka, 1992; Watanabe et al., 1992; Mischak et al., 1993; Li et al., 2003; Martin-Diaz et al., 2007). Therefore, we have a high expectation for the efficacy and safety of DAG-lactone derivatives as novel LRA candidates. As shown in Figures 2, 3, all tested derivatives reversed HIV-1 latency in HIV-1 latently infected cell lines and primary CD4^+^ T cells from HIV-1-infected individuals. Recent studies have highlighted the importance of combining different LRAs from multiple classes (Laird et al., 2015). We also examined the efficacy of DAG-lactone derivatives in combination with a BET inhibitor, JQ1, which reportedly shows synergistic LRA activity with some PKC agonists (Matsuda et al., 2019). Notably, the combination of YSE028 and JQ1 synergistically reactivated HIV-1 latency and exerted potent LRA activity in primary cells from HIV-1^+^ individuals.

We observed an increase in HIV-1 mRNA levels in response to LRAs or PMA (Figure 3). In this regard, we noticed that some patient cells obtained for the assay did not respond to PMA (data not shown). Possible reasons for this are: (1) we used a relatively smaller number of cells per well compared with previous studies by others and (2) there were a very small number of reservoir cells in patient peripheral blood (all patients were in good condition for a long period of time with cART). In most cases, such primary cells respond not only to PMA, but also to other LRAs. However, in some cases (such as participant 7 in Figure 3), we observed an elevation of HIV-1 mRNA with LRAs, even though the cells did not respond to PMA. The mechanism is unknown, but it is thought that if we use a greater number of cells in the assay, they would react to PMA also. We confirmed that exposure to PMA (100 nM with 2 μM ionomycin) for 24 h did not induce cytotoxicity in primary cells (data not shown); however, it is possible that the agents affected cellular homeostasis, resulting in a different response to PMA/ionomycin in patient-derived infected cells.

As shown in Figure 6B, YSE028 had no toxicity in mice; however, we found that the concentration of the drug in mice did not reach high levels and disappeared rapidly (Figure 6C). In general, the cause(s) of the rapid decline of the compound concentration in the plasma may be due to bioavailability, plasma protein binding, or the specific metabolism of mice. In the present study, we showed that the addition of mouse serum reduced the activity of YSE028 (Supplementary Figure S4), indicating the presence of an enzyme(s) that metabolizes the compound. YSE028 has an ester moiety and is thought to be a substrate for CES1, thus it is rapidly metabolized in mouse blood. The distribution pattern of CES1 in organs varies depending on the animal species (Di, 2019). It is thought that the enzyme is more abundant in mouse plasma than human plasma. Thus, it is possible that the kinetics of DAG-lactone derivatives in human blood may be different than they are in mice. The use of other animal models, such as monkeys, that show relatively similar CES1 distribution patterns to humans (Di, 2019), may be suitable for evaluating the PK profiles of this class of molecules. In addition, a plasma esterase-deficient mouse model recently reported could be useful to assess drug PK without the effect of esterase in mouse plasma (Morton et al., 2005). It should be noted that the PK profile of YSE028 presented in this study is limited to the plasma, and it is also important to evaluate drug concentrations in tissues where HIV-1-infected cells exist and replicate.

Protein kinase C consists of several isozymes and is divided into three subfamilies: cPKC, nPKC, and aPKC. Among them, only cPKC and nPKC isozymes require DAG for their activation. Nomura et al. (2011) previously reported that YSE028 works through PKCδ and induces its translocation from the cytoplasm to cell organelles. The potent PKC agonists PEP005, prostratin, and bryostatin-1 also modulate the PKC pathway, but their activation profiles are different (PEP005, PKCδ; prostratin, PKC α; and θ bryostatin-1, and PKC α and δ; Hampson et al., 2005; Trushin et al., 2005; Mehla et al., 2010). Taken together, it is possible that the activation of nPKC isozymes (δ, ε, η, and θ) may contribute to potent and HIV-1-specific activation, which is considered crucial for future LRA candidates. However, in the present study, at higher concentrations, YSE028 induced global T cell activation (Figure 5A) *in vitro*. Thus, further evaluation and modification of compounds may be needed to obtain HIV-1-specific LRAs.

We previously reported the mechanism of PKC-induced apoptosis in HIV-1 latently infected cells (Hattori et al., 2018; Matsuda et al., 2019). Activation of PKC induces tumor necrosis factor receptor-mediated nuclear factor-*κ*B activation, which induces viral transcription. In addition, the tumor necrosis factor receptor simultaneously induces activation of the caspase signaling pathway. Thus, tumor necrosis factor receptor-mediated nuclear factor-κB activation not only increases the production of viral proteins in cells but also triggers apoptosis induced by HIV-1-related proteins inside the cells. As shown in Table 2; Supplementary Figure S1, no cytotoxicity was observed in the tested DAG-lactone derivatives in HIV-1 uninfected cell lines. This reduced toxicity profile of DAG-lactone derivatives was consistent with the minimal caspase-3 induction observed in these cells (Figure 4). Given the fact that DAG-lactones strongly activate caspase-3 in HIV-1 latently infected cells, it is likely that DAG-lactone derivatives kill and eliminate HIV-1 latent reservoir cells more specifically than they do HIV-1 uninfected cells.

In the current well-controlled cART era, neuronal disorders are becoming more frequent in aging HIV-infected individuals because of their long lifespans (Thakur et al., 2019). Notably, HIV-1-associated neurocognitive disorders, which also occur in young people in their 20s and 30s, are a serious problem for HIV-1 infected individuals. HIV-1 invades the central nervous system through the migration of infected monocytes and is subsequently transmitted to various types of cells expressing CD4, including microglia, astrocytes, and perivascular macrophages (Cosenza et al., 2002; Rothenaigner et al., 2007). HIV-1-infected cell populations are established and exist as viral reservoirs (or so-called sanctuaries) in the brain (Hellmuth et al., 2015). The infected microglia and astrocytes release neurotoxicity factors, such as cytokines and chemokines, which disrupt the blood-brain barrier (Gonzalez-Scarano and Martin-Garcia, 2005). HIV-1 proteins, such as gp120, Tat, Vpr, and Nef, also induce inflammation and neuronal apoptosis and trigger neurodegenerative disorders (Canet et al., 2018). Aging is a primary risk factor for neurodegenerative disorders, such as Alzheimer’s disease, which is believed to be caused by similar cellular pathways as HIV-1-associated neurocognitive disorders. Cho et al. (2017) reported that expression of viral proteins, such as gp120, Nef, and Tat, significantly increased the number and size of amyloid plaques in the cerebral cortex of an HIV-1 transgenic rat model. As a novel therapeutic approach for Alzheimer’s disease, PKC activation with PKC agonists (such as benzolactam or bryostatin-1), is thought to be effective in reducing amyloid beta formation *via* the promotion of α-secretase production, which leads to cleavage of the amyloid precursor protein (Etcheberrigaray et al., 2004). Although we did not evaluate the regulation of amyloid beta by PKC agonists in this study, activation by DAG-lactone derivatives (which utilize similar PKC isozymes as bryostatin-1) may also contribute to it. In fact, Lee et al. (2006) reported that DAG-lactone potently induced α-secretase activation, which resulted in the reduction of amyloid beta peptide deposition. Further investigations are needed to clarify the effect of PKC agonists, including DAG-lactones, on HIV-1-associated neurocognitive disorders and other similar conditions.

In summary, DAG-lactone derivatives, including YSE028, are potential candidates for novel therapeutics to combat HIV-1 reservoirs in HIV-1-infected individuals. However, even though these compounds have been confirmed to be safe in the animal models, more detailed *in vitro* and *in vivo* analyses are necessary to accumulate information regarding the efficacy of these drugs on a wide variety of HIV-1 reservoir cells that hide and exist in many organs *in vivo*.

## Data Availability Statement

The datasets presented in this study can be found in online repositories. The names of the repository/repositories and accession number(s) can be found in the article/Supplementary material.

## Ethics Statement

The studies involving human participants were reviewed and approved by The Ethics Committee at the National Center for Global Health and Medicine. The patients/participants provided their written informed consent to participate in this study. The animal study was reviewed and approved by Institutional Animal Care and Use Committee at Kumamoto University.

## Author Contributions

KeM and KoM designed and performed the experiments and wrote the manuscript. TK and HT synthesized the compounds. RK, SR, and SO performed the experiments. KiT, KoT, TI, HG, KY, AH, HM, and HT provided suggestions for the experimental design. All authors contributed to the article and approved the submitted version.

### Conflict of Interest

The authors declare that the research was conducted in the absence of any commercial or financial relationships that could be construed as a potential conflict of interest.

## Supplementary Material

Supplementary Figure S1 Cell viabilities of A3.01 **(A)** and U937 **(B)** cells exposed to different concentrations of diacylglycerol (DAG)-lactone derivatives. Cell viabilities were determined using the 3-(4,5-dimethylthiazol-2-yl)-s,5-diphenyltetrazolium assay on day 7. **(C)** Acute cytotoxicity of protein kinase C (PKC) activators including YSE028 in primary cells. PBMCs from three healthy donors were exposed to different concentrations of a reagent for 24 h. The ratio of living cells was calculated for fixable viability dye negative cell populations by flow cytometry. Data are shown as means ± SDs of three independent experiments.

Supplementary Figure S2 Expression of intracellular HIV-1 p24 proteins in ACH-2 **(A)** and U1 **(B)** cells. The cells were exposed to different concentrations of DAG-lactone derivatives, and intracellular p24 levels were examined by flow cytometry analysis after 24 h.

Supplementary Figure S3 **(A)** Toxicity of YSE028 in primary cells. **(B)** Changes in CD69 expression on CD4^+^ or CD8^+^ primary T cells after exposure to combined latency-reversing agents (LRAs) for 24 h were analyzed by flow cytometry. Data are shown as means ± SDs.

Supplementary Figure S4 J-Lat 10.6 cells were exposed to YSE028 or prostratin for 24 h in the presence of 10% fetal bovine serum **(A)** or 10% mouse serum **(B)**. The number of green fluorescent protein (GFP)-positive cells was analyzed after 24 h by flow cytometry. Data are shown as means ± SDs of three independent experiments.
