# Supplementary figures and images for: A Therapeutic Strategy to Combat HIV-1 Latently Infected Cells With a Combination of Latency-Reversing Agents Containing DAG-Lactone PKC Activators
Source: Front Microbiol. 2021 Mar 17;12:636276. doi: 10.3389/fmicb.2021.636276 (PMC8010149; doi:10.3389/fmicb.2021.636276)

Supplemental Figure.1

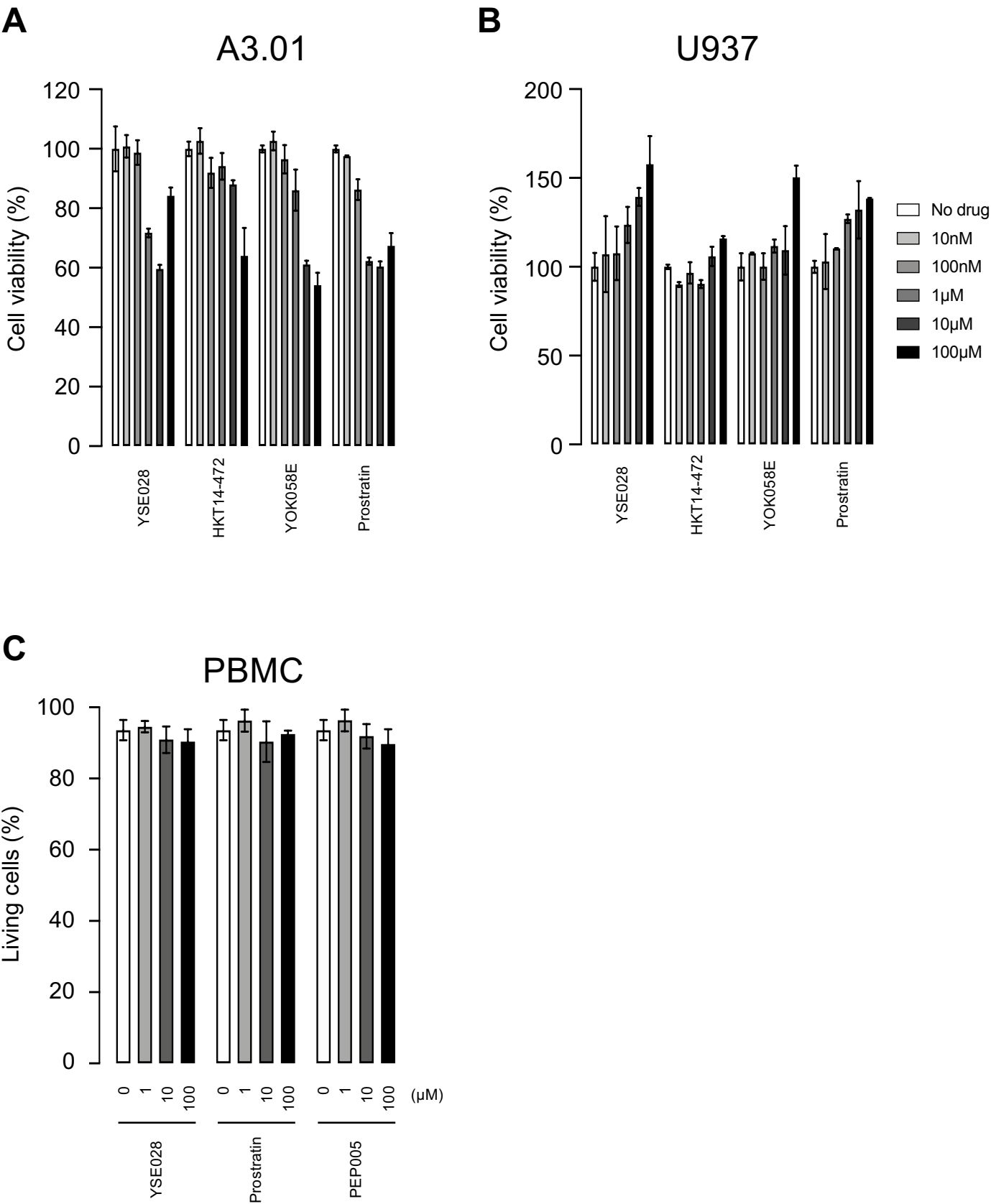

Supplemental Figure.2

A

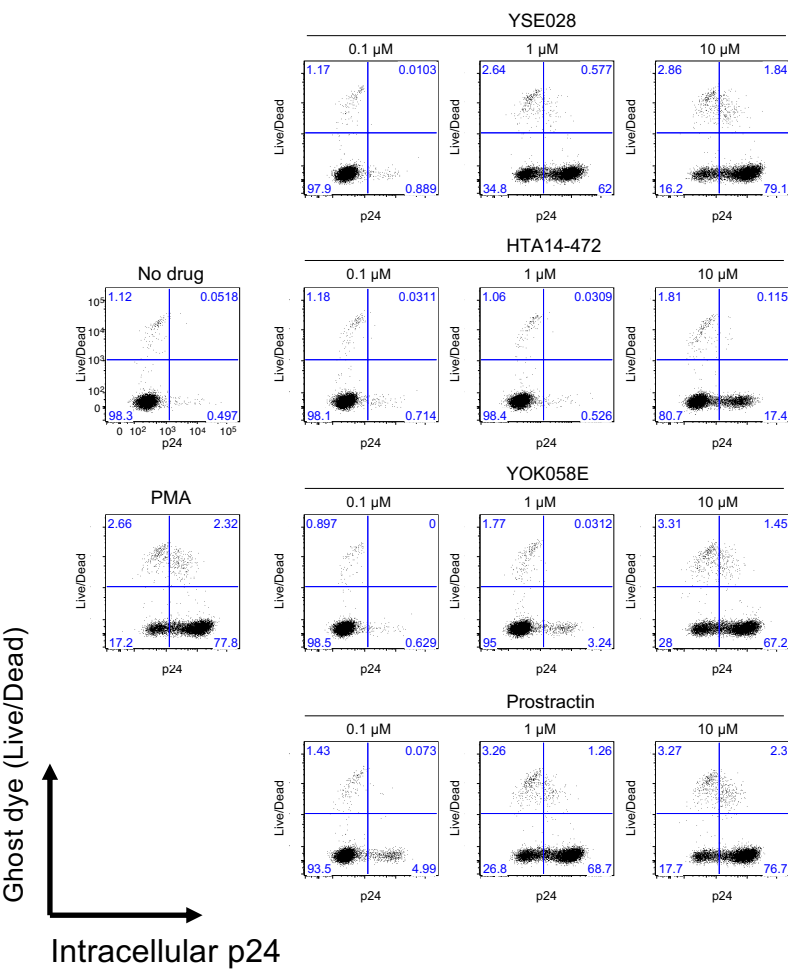

B

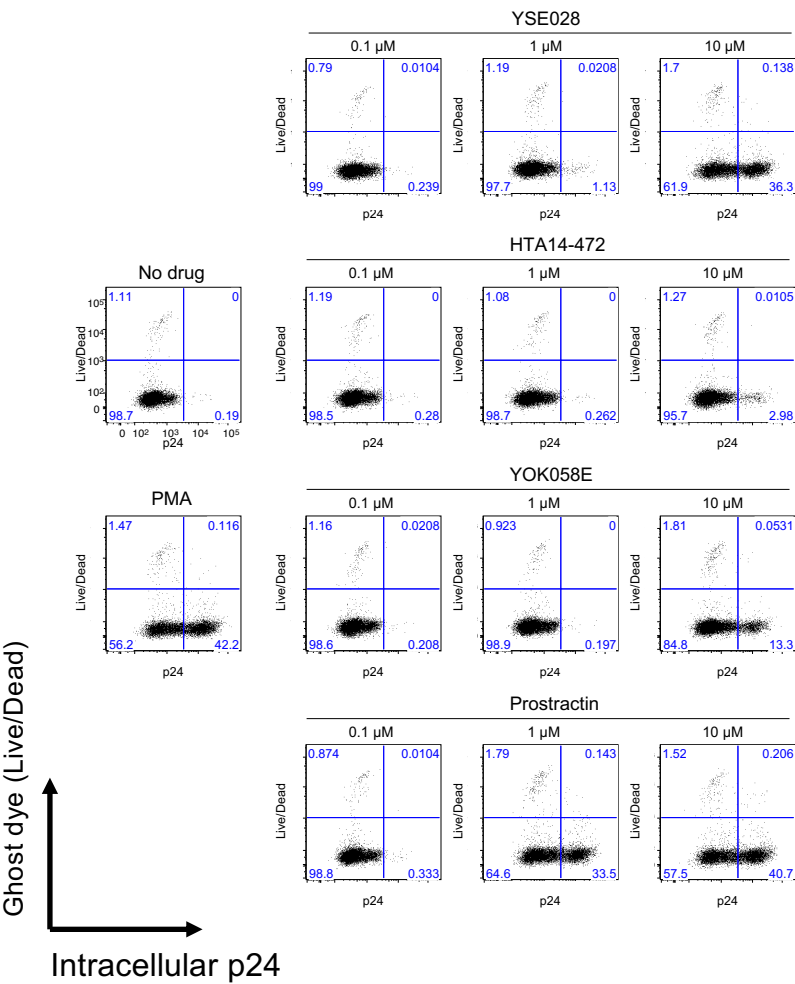

Supplemental Figure.3

A

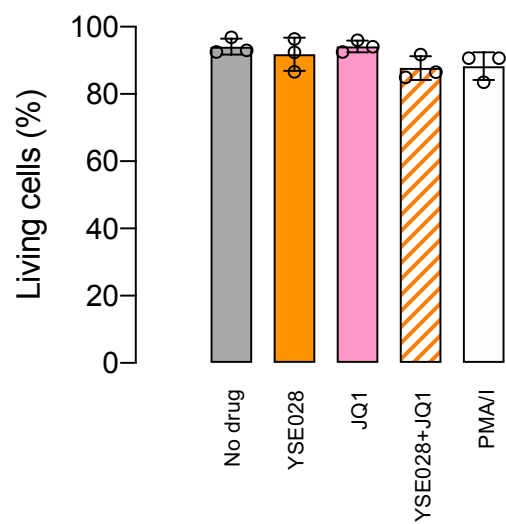

B

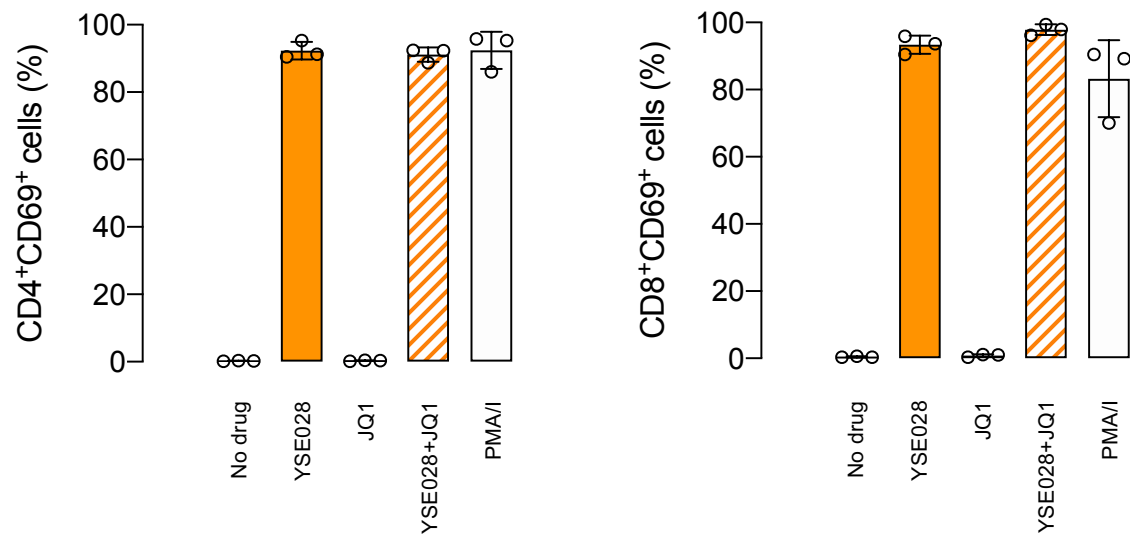

Supplemental Figure.4

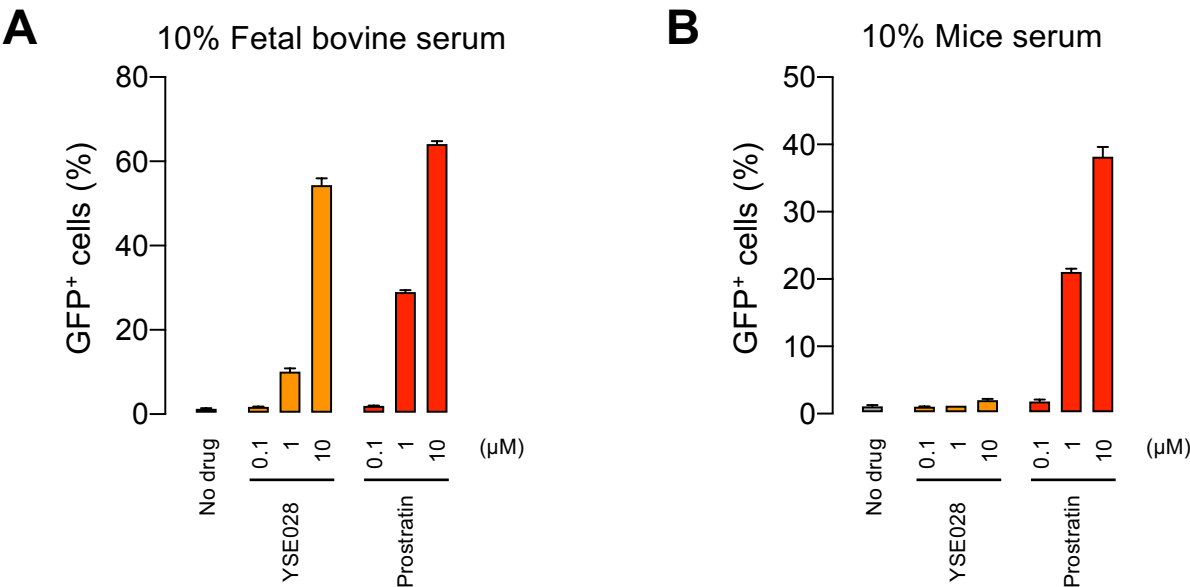

Supplement: Supplementary Figure S1 — Cell viabilities of A3.01 (A) and U937 (B) cells exposed to different concentrations of diacylglycerol (DAG)-lactone derivatives. Cell viabilities were determined using the 3-(4,5-dimethylthiazol-2-yl)-s,5-diphenyltetrazolium assay on day 7. (C) Acute cytotoxicity of protein kinase C (PKC) activators including YSE028 in primary cells. PBMCs from three healthy donors were exposed to different concentrations of a reagent for 24 h. The ratio of living cells was calculated for fixable viability dye negative cell populations by flow cytometry. Data are shown as means ± SDs of three independent experiments. [file Data_Sheet_1.pdf]
